# Poultry disease occurrences and their impacts in Ethiopia

**DOI:** 10.1007/s11250-020-02465-6

**Published:** 2021-01-03

**Authors:** Yohannes T. Asfaw, Gobena Ameni, Girmay Medhin, Balako Gumi, Yohannes Hagos, Barbara Wieland

**Affiliations:** 1 International Livestock Research Institute (ILRI), P.O. Box: 5689, Addis Ababa, Ethiopia; 2 Aklilu Lemma Institute of Pathobiology, Addis Ababa University, P. O. Box: 1176, Addis Ababa, Ethiopia; 3 College of Veterinary Sciences, Mekelle University, P. O. Box: 8024, Mekelle, Ethiopia; 4 Department of Veterinary Medicine, College of Food and Agriculture, United Arab Emirates University, P.O. Box 15551, Al Ain, UAE

**Keywords:** Poultry diseases, Poultry diseases impact, Ethiopia

## Abstract

Poultry production contributes significantly to the livelihoods of Ethiopian farmers and to the national economy although it is hampered by different factors, including poultry diseases. There is scarcity of published evidences on the occurrence and impacts of poultry diseases although such evidences are important for policy makers in designing appropriate interventions. A total of 595 households were interviewed and 11 FGDs were conducted to collect data on the occurrence of diseases and the number of dead chickens in the last 12 months. Hence, respiratory diseases, sudden death, and eye-face-head diseases were mentioned in all of the FGDs as the most frequently occurring disease in the districts. Of households interviewed, 86.1% reported poultry disease occurrence in the last 12 months, and gastrointestinal, eye-face-head, and neurological diseases were identified to be the top three ranked diseases of chickens in the districts. Flocks with access to diagnostic services (Adj. OR = 4.16; *P* = 0.004) and/or access to animal health providers (Adj. OR = 10.50; *P* = 0.001) were more likely to report disease occurrence. In the studied population, the diseases resulted in deaths of 2219 chickens valued at 352,219.5 Birr (11,740.65 USD) and a mean crude mortality of 31.87%. Female-lead households (mean difference = 5.95%; *P* = 0.018) and multiple age units present on the farm (mean difference = 20.92%; *P* = < 0.000) had higher chicken mortality. Similarly, higher mortality was reported in flocks without access to diagnosis (mean difference = 9.97%; *P* = < 0.000) and vaccination (mean difference = 12.34%; *P* = < 0.000) services. The high occurrence of disease and mortalities might be explained by a lack of an organized poultry health service delivery system in the country. Therefore, a carefully designed health service delivery system addressing needs of poultry producers, supported by relevant policy and corresponding strategies, is recommended to address the indicated challenges. Moreover, private health providers with well-defined roles need to be engaged to successfully and sustainably solve the poultry disease problems.

## Background

Poultry in Ethiopia are potentially an excellent animal protein supplier serving as an important contributor for food and nutrition security, and they are sources of cash income for a large part of the population (Shapiro et al. 2015; Wubet et al. 2019). However, there is lack of adequate scientific information on occurrences and impacts of poultry diseases in the country, which could have negative implications on establishing a sustainable and profitable poultry business in the country.

Poultry diseases are responsible for a number of adverse economic and social impacts. Their occurrence depends on various factors including geo-climatic condition, population density, management practices, and immunization status (Al Mamun and Mehetazul 2019). They lead to high mortality and morbidity of chickens, high medication costs, loss in production and market, and can pose a risk to public health through zoonoses (Wubet et al. 2019). Hence, poultry disease status, poultry morbidity, and mortality are useful measurable indicators to judge the overall health status of a flock and its productivity (Marangon and Busani 2007). These indicators can also be used to monitor performances of interventions designed for disease control and prevention. Disease outbreaks were recently reported as major constraints of poultry producers (Ebsa et al. 2019) and high chick mortality caused by disease and predation in Ethiopia has been reported earlier (Habte et al. 2017). Newcastle disease (ND), salmonellosis, fowl cholera, coccidiosis, and fowl pox were reported as main infectious diseases causing high morbidity and mortality both in village and in large-scale poultry farms (Wubet et al. 2019).

Higher mortality of chickens in northwest Ethiopia because of improper nutrition, substandard hygienic conditions, lack of appropriate disease prevention, and control program has compromised their expected contribution to household livelihoods (Mazengia et al. 2012). On the other hand, an overall chicken mortality of 26.3% in three commercial poultry farms in central Ethiopia was reported (Chanie et al. 2009).

Similarly, estimates of overall mortality (56.5%) and morbidity (58.1%) were reported by the Central Statistics Agency of Ethiopia (2018) in peasant-owned chicken flocks only. However, these estimates may not reflect the actual situation on the ground because the CSA data are of estimate data collected and analyzed by non-veterinary researchers and without adequate consultations of the poultry owners. Hence, use of these data may not be ideal to inform policy makers and design cost-effective poultry disease prevention and control interventions.

While previously conducted studies highlighted the high negative impact of disease, there is a clear lack of evidence that quantifies the overall status of poultry disease occurrence, and chicken morbidity and mortality in Ethiopia that can be used by policy makers. Hence, except for a few efforts which are implemented in response to outbreaks, almost no strategic poultry health interventions are being designed and implemented in the country to reduce the negative impacts of poultry diseases and other health problems in the poultry sub-sector. This, in turn, has led to difficulty to achieve the ambitious targets of the poultry sub-sector (Shapiro et al. 2015). Some of the ambitious targets of the Ethiopian Government’s poultry sub-sector masterplan include transformation of the traditional family poultry production to improved family poultry production, and a 247% and 828% increase in chicken meat and egg production by 2020, respectively (Shapiro et al. 2015).

Hence, this study was conducted to generate evidences on the magnitude of disease occurrence, chicken’s mortality, and examine the impacts of possible factors on these outcomes and their monetary impact.

## Materials and methods

### Study area and study population

**Fig. 1:**
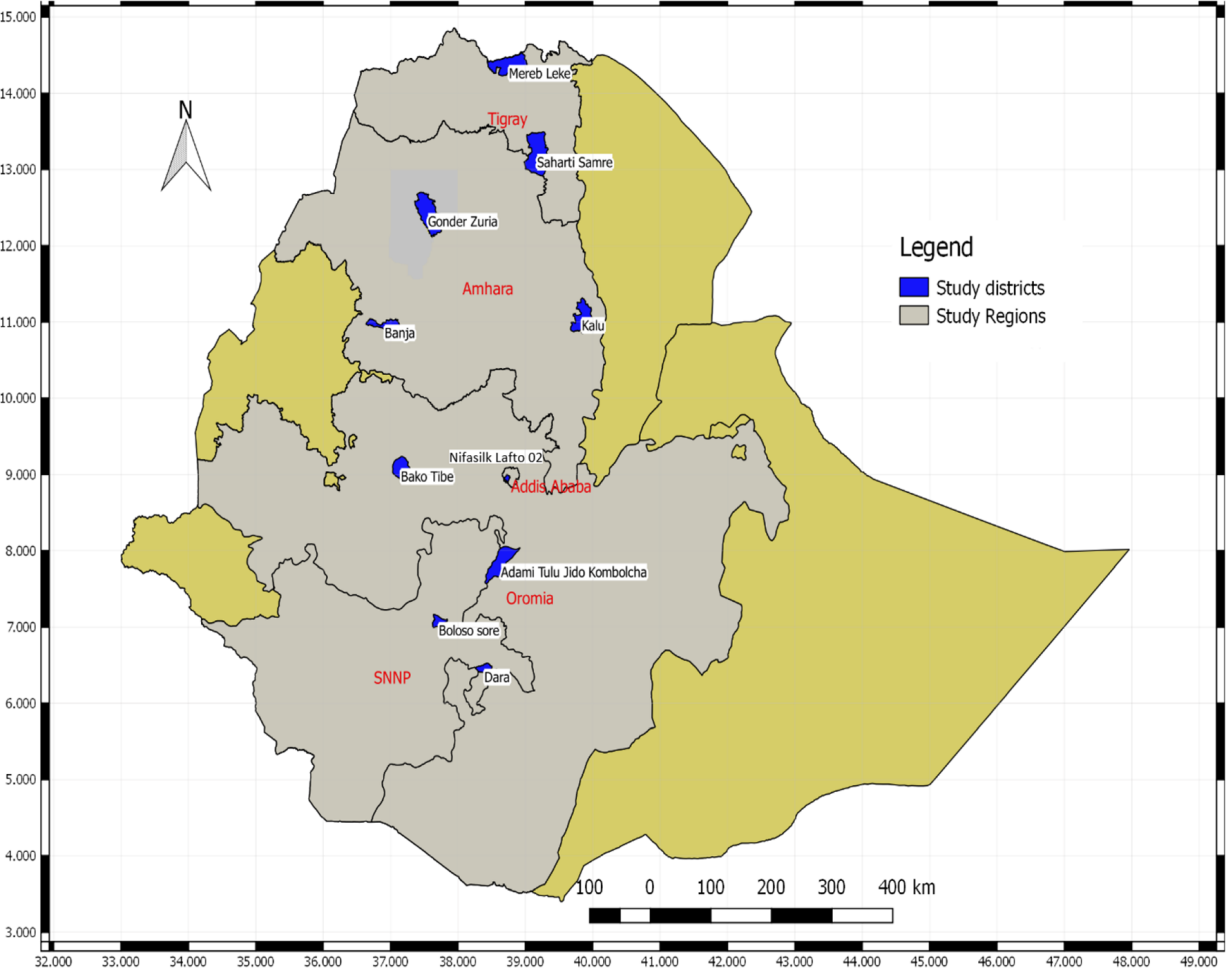
Map of Ethiopia with the 10 study districts which were included in the survey. Source: Produced by Author, 2019

The study was conducted in four regions (Oromia, Amhara, SNNP, and Tigray) and Addis Ababa city administration which include the following 10 districts: *Sahrti Samre* and *Mereb Leke* from Tigray Region, *Banja Shekudad* Gondar *Zuria* and *Kalu* from Amhara Region, *Bako Tibe* and *Adami Tulu* from Oromia Region, *Dara* and *Boloso Sore* from Southern Ethiopia Nations, Nationalities and People’s (SNNP) Region, and *Nifasilk Lafto* from Addis Ababa City Administration (Fig. 1). In the ten study districts, a total of 29 *kebele* (*Kebele* is the smallest Government administration unit organized under each district) were included the survey.

The source population/study population were all poultry-owning households/chicken owners enrolled in the African chicken genetics gain (ACGG) project implemented by the International Livestock Research Institute (ILRI) in four regions and one city administration of Ethiopia. Hence, households that reared chickens of all age groups, both sexes, and all breeds managed under extensive and semi-intensive production systems in the selected districts were eligible to be the target populations.

### Study design and study period

The study was conducted from 2018 to 2019 using a cross-sectional household questionnaire survey and focused group discussion study designs.

### Sample size and sampling procedures

As per the sampling framework of the ACGG project (ILRI-ACGG 2015), twenty-two districts from four out of total nine regions and Addis Ababa city administration were considered to represent high chicken rearing potential of the country in the ACGG project, as they were selected based on preset criteria. The criteria were set by workshop participants who came from different parts of the country and who are knowledgeable in poultry production of the country. The criteria used by the project to select the regions, districts, and *Kebele* were number of chicken, number of (smallholder) households rearing chicken, percent contribution of chicken to household income/nutrition, percent market share captured by smallholder poultry farmers, availability of feed resources for a growing chicken industry, and fair diversity in terms of agro-ecology and geographical locations across the regions. Moreover, other criteria such as cost-effectiveness, accessibility, security concerns, distance from Addis Ababa (Capital city of Ethiopia), and others such logistical issues were also used after the regions were ranked using the abovementioned criteria. According to the above criteria, four regions, one city administration (Addis Ababa), and 22 districts from the designated regions and city were selected. Then, the kebele (village as per ACGG) were randomly selected from the selected 22 districts considering agro-ecological situations within a district and accessibility as additional criteria.

For this study, one up to three district(s) was/were randomly selected from each of the four regions (Oromia, Amhara, SNNP, and Tigray) and Addis Ababa city administration (Fig. 1). Accordingly, two to three Kebele per the designated district were randomly selected from the ACGG beneficiary Kebele found in the district. Finally, the chicken-owning households/chicken owners found in each of those Kebele were then randomly selected. Replacement for unwilling randomly selected households was replaced by their neighbors who agreed to participate in the survey.

Hence, EpiTools epidemiological calculators as per Sergeant (2009) and Charan and Biswas (2013) was used to calculate sample size for the cross-sectional survey. Accordingly, the total sample size for the 10 districts was 595 (approximately 60 participants per district), considering 50% estimated proportion of chicken flocks with history of disease or chicken mortality, 5% desired precision, and 95% confidence level.

Moreover, in each study district except one, a focus group discussion (FGDs) was conducted, with 10–15 participants who were purposively selected using preset criteria. The tenth FGD was not conducted due to security issue. The FGD participants were selected from chicken farmers who own five and above chickens, are reported to have basic knowledge on poultry diseases and their impacts, willing to participate, and were ACGG project beneficiaries. Moreover, gender, age, and geographic representations of the discussants in each district were also used as additional preset criteria.

### Data collection

A semi-structured questionnaire was developed for the purpose of the current survey. Enumerators working for the ACGG project in each of the study Kebele were recruited and trained to serve as data collectors. The questionnaire was translated into local languages (Tigrigna, Amharic, Oromiffa). Then, the developed questionnaire was pre-tested in non-participating household equivalent to 4% of the total sample size, to evaluate its logical flow and time it takes for the interview. Appropriate modifications were made on the questionnaire based on the feedback from the pre-testing. The questionnaire captured data on disease occurrence, mortality in the flock, geographic location (Region and District), demographic characteristics of respondents, flock characteristics, production system, health service parameters, and farm biosecurity measures, and included questions about critical challenges of the poultry health service delivery. All respondents were asked to give oral consent before the interview and were encouraged to freely respond to the questions included in the questionnaire.

Nine focused group discussions (FGD), one in each of the districts except Bako Tibe district, were held to generate first-hand and detailed information on disease occurrence and chicken death, to support the findings of the questionnaire survey. The FGD participants were farmers of both sexes selected by ACGG field workers in consultation with livestock experts of each district, based on their capability of answering questions related to poultry diseases. The discussions were facilitated and monitored by the researcher and a checklist guided the sequence of information to be collected from the focus group discussions. Participants were then asked to discuss the overall challenges they face in their poultry flocks. Then, they were asked to discuss on status of disease occurrences and describe major clinical signs observed during the occurrence of the disease and chicken deaths.

### Definition of disease occurrence

Household survey participants were asked whether their chicken had been sick in the last 12 months prior to the interview and to describe the main disease symptoms they observed on the sick chickens. The respondents were also asked to rank the disease symptoms they observed into five-point Likert scale (very important, important, so-so, less important, or not important). Then, the disease occurrence was defined as “yes” responses when respondents answered “yes” and were able to mention and rank the following disease symptoms. The pre-defined disease symptoms were sudden death, general weakness (lethargy) expressed by not eating and moving, dull and/or closed eyes, abnormal discharges (nasal, ocular), abnormal droppings/diarrhea, ruffled/loss of feathers, skin lesions, paralysis (neck twisting, leg, wing paralyzes), abnormal sitting (sitting on haunches or lying down), wing droppings, discolorations (head, wattle, comb) and/or staining (vent), weight loss, sudden production lose/egg laying, abnormal gait/lameness, and/or any local swelling and related symptoms. Then, the described disease symptoms were grouped into disease problem categories, which were gastrointestinal, eye-facial-head, respiratory, reproductive, neurological, musculoskeletal problems, and general illness as well as sudden deaths and parasite infestation. Eye-facial-head problems are defined as any disease manifestations as reported by the respondents and which locally affect either eye, face, or head parts of the chickens.

### Crude mortality and associated monetary loses

Crude mortality was defined as the total number of dead chickens due to any disease that occurred over 12 months prior to the interview divided by the total flock size and then, multiplied by 100, i.e., CM = *n*/N*100. CM—crude mortality, *n*—total number of dead chickens, and *N*—total flock size. The flock size was considered to be the flock size reported during the survey plus the number of dead chickens over the 12 months prior to the interview. It was assumed that the numbers of exit and entry of chickens to the flock are comparable, because 80% of the chicken farmers keep 2–9 chickens in a flock (FAO 2019).

Monetary loss due to crude mortality was defined as the monetary value of the total number of dead chickens due to any disease over 12 months prior to the interview. To calculate the monetary lose, the total number of dead chickens was converted into monetary value upon multiplying it by the average live chicken market price of the same period, i.e., 158.8 ETB. The average live chicken market price was calculated considering the prices of all age groups (Hagos 2019).

### Data management and analysis

The raw data generated from the survey were entered into Microsoft Excel and after checking data consistency, exported into STATA (Version 14, StataCorp, USA) for analysis. A priority index was used to rank the disease problems based on their relative importance using the following formula as described by Musa et al. (2006). Priority index (PI) = (*F*1*3) + (F2*2) + (*F*3*1)/FT, where *F*1—frequency of the first rank, *F*2—frequency of second rank, *F*3—frequency of third rank, FT—Frequency of total respondents. The associations between the explanatory variables and the outcome variables (disease occurrence and crude mortality) were further assessed using chi-square test and a pairwise correlation matrix. All independent variables which were significantly associated with each of the outcome variables at *P* < 0.05 were considered for regression analysis.

Binary logistic and linear regression models were built using a step-wise model building approach to detect associations of independent variables with the binary (disease occurrence) and continuous outcome variables (crude mortality), respectively. Goodness of fit of logistic regression model was assessed using a Hosmer-Lemeshow goodness-of-fit test. Similarly, model fit of multiple linear regression was checked using specificity, multicollinearity, normality, and homoscedasticity tests. Finally, the results of the influences were reported in the form of adjusted odds ratios for binary outcomes and mean difference for continuous outcomes. For all analyses, a *P* value < 0.05 was used as cut-off point for significance.

### Ethical considerations

The study was ethically approved by the Addis Ababa University Aklilu Lemma Institute of Pathobiology’s institutional ethical review board (Ref. No.: ALIPB/IRB/007/2017/18) and written or oral consents from study participants were obtained.

## Results

### Overall disease occurrence and effects of associated predictor variables

A total of 113 poultry farmers, of which 58 females, participated in the FGDs. During the FGDs, the occurrence of poultry disease was well acknowledged by all of the discussants and they boldly quoted it as **“Disease is an invisible enemy of their chickens.”** Moreover, some discussants while expressing the impact of poultry diseases they stated it as **“Should our chickens be free from disease, we would have been profitable.”**

**Fig. 2:**
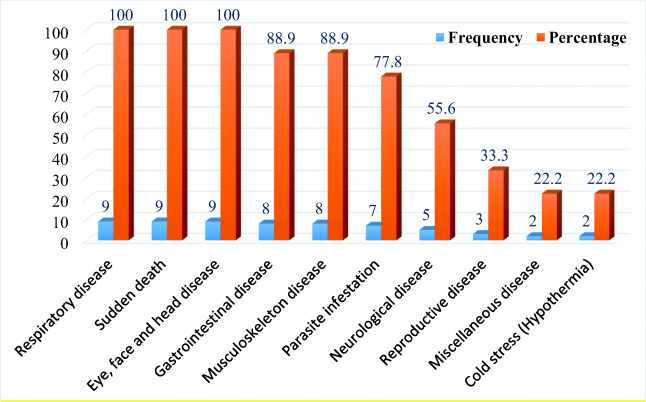
Frequency of the poultry disease problems mentioned during the FGDs

They reported high disease occurrences in the majority of the chicken flocks found in the study districts. All of the FGD discussants witnessed clinical symptoms of respiratory diseases, sudden death, and eye-face-head diseases as three most frequently occurring disease problems to their chicken flocks (Fig. 2). On top of this, the reported disease symptoms were considered to be suggestive of infectious diseases such as Newcastle disease, avian coccidiosis, fowl pox, fowl cholera, and salmonellosis. The FGD discussants ensured that they identify Newcastle disease and fowl pox disease by their vernacular names “*Fengil*” and “*Fentata*,” respectively.

In the questionnaire survey, a majority of households (86.1%, 470/546) witnessed disease occurrence in their chicken flocks over the 12 months before the survey (Tables 3). Categories of nine different disease problems were identified to have occurred (Table 1). Using index analysis, the identified disease problems were ranked. The index scores for the top five ranked diseases of poultry were 0.70 for gastrointestinal disease, 0.60 for eye-facial-head disease, 0.44 for sudden death, 0.27 for respiratory disease, and 0.26 for neurological disease (Table 1).

**Table 1:** Prioritized categories of disease problems and their respective clinical signs as reported by farmers

Categories of disease	System-specific disease symptoms as reported by respondents	Frequency of responses for the disease categories					Priority indexes (PI)	Rank
		1st	2nd	3rd	4th	5th		
Gastrointestinal disease	Different types of diarrhea, staining of vent hairs, floor wetting with or without deaths	116	66	50	28	6	0.70	1
Eye, facial and head diseases	Abnormal discoloration and swelling of wattle and comb; swelling and inflammation of eye, face, and/or head; ocular discharge; deaths	105	51	38	23	19	0.60	2
Unidentified sudden deaths	Sudden death of chickens without showing overt disease symptom	124	0	0	19	7	0.44	3
Respiratory disease	Coughing, nasal discharges, breathing difficulty, gasping (mouth breathing), abnormal respiratory sound, and/or deaths	57	0	0	52	19	0.27	4
Neurological disease	Neck, leg, and/or wing paralysis, incoordination, head trembling, loss of nerve sensation, and/or death	66	0	0	27	5	0.26	5
General illness	Dullness, depression, unable to eat and move around, weightlessness, closed eye, and with no system-specific symptoms	39	0	0	4	1	0.14	6
Reproductive disease	Egg deformities such as thin or without shell, prolapse of reproductive organ, not laying egg (infertility)	23	0	0	8	5	0.09	7
Musculoskeletal disease	Presence of visible injury, swelling and deformities on muscle, skin, bone, legs, and associated parts	14	0	0	3	7	0.05	8
Ecto-parasite infestations	Infestation with different ecto-parasites such as of mite, lice, flea, tick, restlessness, itching, stunting, anemia	3	0	0	0	0	0.01	9
Total		547	117	88	164	69		

NB: *1st* more frequent, *2nd* frequent, *3rd* so-so, *4th* less frequent, *5th* not frequent

In addition, 44.7% and 12.8% of the reported cases assigned to the gastrointestinal health problem category were bloody diarrhea in young chickens indicating, e.g., coccidiosis, Newcastle disease, and other gastrointestinal disease and white diarrhea sticky to vent suggesting pullorum disease. Similarly, 19.5% of the cases recorded in the eye-facial-head disease category were open and scabby type of wound on featherless body of the chickens indicating fowl pox where farmers also identified this disease by its vernacular name “*Fintata*,” while 27.5% were red brown discoloration and swelling of wattle and comb with deaths being suggestive of fowl cholera.

The highest disease occurrence rates was reported for SNNP region (94.64%), Dara District (98.21%), flocks with a size of less than 5 chickens (92.67%), chicken flocks which had access to diagnosis (92.82%), diagnosed by animal health experts (95.93%), vaccinated by non-animal health experts (91.07%), did not to receive treatment (91.07%), and those that received ineffective treatment (91.43%).

Multivariable analysis to identify factors that significantly influence occurrence of disease showed that chicken flocks with access to diagnostic services (adjusted odds ratio = 4.16; 95%CI: 1.57–11.00), chicken flocks reported to be diagnosed by animal health experts (adjusted odds ratio = 10.50; 95%CI: 2.83–38.96), and chicken flocks reported to have been vaccinated by non-animal health experts as compared to professional animal health service providers (adjusted odds ratio = 3.50; 95%CI: 1.37–8.97) had higher odds to report disease occurrence in the survey (Table 2).

**Table 2:** Disease occurrence stratified by selected characteristics and results of logistic regression analysis of the effects of independent variables

Predictor variables		Disease occurrence		Crude odds ratio (95% CI)	Adjusted odds ratio (95% CI)	*P* value
		*N*	Yes (%)			
Region	Tigray	108	90(83.33)	1.15(0.57–2.32)	0.29(0.04–2.19)	0.229
	Amhara	163	137(84.05)	1.21(0.64–2.30)	0.37(0.05–2.47)	0.303
	Oromia	107	87(81.31)	1.92(0.72–0.09)	0.37(0.04–3.42)	0.381
	Addis Ababa	56	50(89.29)	4.06(1.56–10.56)	0.96(0.15–6.05)	0.968
	SNNP	112	106(94.64)	ref	Ref	-
District	Mereb leke	56	48(85.71)	2.31(0.89–6.01)	2.90(0.45–18.68)	0.262
	Sahrti Samre	52	42(80.77)	1.62(0.65–4.02)	-	-
	Gondar Zuria	57	49(85.96)	2.36(0.91–6.13)	1.31(0.23–7.50)	0.764
	Banja *Shekudad*	52	49(94.23)	1.96(0.25–15.43)	1.96(0.25–15.43)	0.519
	Kalu	54	39(72.22)	ref	Ref	-
	Bako	54	42(77.78)	1.35(0.56–3.23)	-	-
	Adami Tulu	53	45(84.91)	2.16(0.83–5.65)	7.16(0.60–85.25)	0.119
	*Nifasilk Lafto*	56	50(89.29)	3.21(1.14–9.03)	-	-
	Bolose Sore	56	51(91.07)	3.92(1.31–11.72)	-	-
	Dara	56	55(98.21)	21.15(2.68–66.87)	-	-
Flock size	≤ 5 chickens	150	139(92.67)	2.79(1.24–6.26)	1.30(0.22–7.72)	0.772
	6–10 chickens	151	130(86.09)	1.37(0.68–2.75)	0.86 (0.19–3.96)	0.849
	11–20chickens	132	106(80.30)	0.90(0.45–1.77)	1.64 (0.41–6.67)	0.484
	> 20 chickens	94	77(81.91)	ref	Ref	-
Access to disease diagnosis	Yes	209	194(92.82)	2.22(1.20–4.10)	4.16(1.57–11.00)	0.004
	No	307	262(85.34)	ref	ref	
Diagnosis made by	Animal health expert	123	118(95.93)	4.81(1.85–12.47)	10.50(2.83–38.96)	0.001
	Non-animal health experts	254	211(83.07)	ref	Ref	
Access to treatment	Yes	219	180(82.19)	5.54(3.31–9.28)	1.81(0.66–4.93)	0.248
	No	280	255(91.07)	ref	Ref	
Effectiveness of treatment given	Good effectiveness	158	127(80.38)	ref	Ref	-
	Fairly effective	266	236(88.72)	2.60(1.03–6.56)	2.39(0.54–10.53)	0.250
	Not/Ineffective	70	64(91.43)	1.92(1.11–3.32)	2.34(0.83–6.63)	0.110
Types of chicken vaccinators	Animal health expert	219	180(82.19)	ref	Ref	0.009
	Non-animal health experts	280	255(91.07)	2.21(1.29–3.78)	3.50(1.37–8.97)	

### Crude chicken mortality and effects of associated predictor variables

The overall crude chicken mortality due to diseases was 31.87% (95%CI: 29.33–34.41). Higher chicken mortalities were reported in Adami Tulu District (mean = 43.18%; 95%CI: 33.85–52.50), of female respondents who owned flocks (mean = 37.92%; 95%CI: 33.94–41.90), flocks with multiple age groups (mean = 50.39%; 95%CI: 44.37–56.42), chickens kept in extensive farming system (mean = 34.96%; 95%CI: 31.66–38.27), chicken flocks without access to diagnosis service (mean = 39.47%; 95%CI: 36.08–42.86) and chicken flocks without access to vaccination (mean = 45.14%; 95%CI: 39.58–50.70) as compared to their respective categories (Table 3).

**Table 3:** Overall and variable-specific means and results of multiple variables linear regression analysis for crude mortality

Predictor variables		Crude morality		Linear regression		*P* value
		Mean	(95% CI)	Mean difference	(95% CI)	
District	*Mereb leke*	30.72	22.68–38.75	− 4.36	− 15.76–7.04	0.45
	*Sahrti Samre*	38.66	29.55–47.77	− 1.87	− 14.19–10.44	0.77
	Gondar *Zuria*	32.60	25.59–39.61	− 6.15	− 17.48–5.18	0.29
	*Banja Shekudad*	29.79	22.17–37.42	− 5.83	− 17.05–5.38	0.31
	*Kalu*	28.54	21.55–35.53	− 8.20	− 19.23–2.83	0.15
	*Bako*	26.01	20.03–31.99	− 9.71	− 20.83–1.42	0.09
	*Adami Tulu*	43.18	33.85–52.50	8.79	− 3.51–21.08	0.16
	*Nifasilk Lafto*	33.20	24.45–41.95	− 2.55	− 14.51–9.40	0.68
	*Dara*	20.14	14.42–25.86	− 9.21	− 20.07–1.64	0.09
	*Boloso Sore*	36.47	26.92–46.02	Ref	Ref	Ref
Sex of respondents	Male	27.19	24.00–30.38	Ref	Ref	Ref
	Female	37.92	33.94–41.90	5.95	1.03–10.88	0.018
Age-group of flocks	Single age unit	26.45	23.88–29.02	Ref	Ref	Ref
	Multiple age unit	50.39	44.37–56.42	20.92	14.12, 27.72	0.000
Production system	Extensive	34.96	31.66–38.27	5.15	0.59–9.72	0.027
	Modern	25.91	22.17–29.66	Ref	Ref	Ref
Access to diagnosis service	Yes	23.19	19.50–26.88	Ref	Ref	Ref
	No	39.47	36.08–42.86	9.97	5.16–14.79	0.000
Access to vaccination	Yes	27.23	24.49–29.97	Ref	Ref	Ref
	No	45.14	39.58–50.70	12.34	6.37–18.32	0.000
Total		31.87	29.33–34.41		0.528	

Multiple linear regression analysis found that the magnitude of mortality reported in female-owned chicken flocks (mean difference = 5.95%; 95%CI: 95%CI: 1.03–10.88%) was higher than the magnitude of mortality in male-owned chicken flocks. Similarly, the mortality in multiple-age-unit chicken flocks (mean difference = 20.92%; 95%CI: 14.12–27.72) was higher than the mortality reported in single-age-unit flocks. Moreover, the magnitudes of mortality in chicken flocks which had no access to diagnosis and vaccination services (mean difference = 9.97%; 95%CI: 5.16–14.79 and mean difference = 12.34; 95%CI: 6.37–18.2, respectively) were higher than the magnitudes of mortality in chicken flocks which had access to diagnosis or vaccination services (Table 3).

The household level annual mean monetary loss attributed to the above-reported chicken mortalities was 592 ETB (95%CI: 535.98–647.95) (19.7 USD). A total of 2219 chickens were reported to have died of the reported disease problems among the surveyed households. Accordingly, the total monetary loss directly attributed to the reported chicken mortalities was calculated to be 352,219.5 ETB which is equivalent to 11,740.65 USD (Table 3). Extrapolating these findings to an estimated 9.85 million number of poultry rearing households in Ethiopia (CSA 2018), losses associated with disease are estimated at 5.8 billion ETB or 194 million USD (CSA 2018) (CSA 2018) (CSA 2018) (CSA 2018).

## Discussion

In both cross-sectional surveys, categories of nine to ten disease problems were identified and prioritized as major causes of chicken illness. The high disease occurrence was reported to cause high crude mortalities, which directly translate into monetary loss of smallholder poultry producers.

This in turn significantly reduces the expected socio-economic returns from the chicken flocks kept by farmers and smallholder poultry producers in the country. The high monetary loss attributed to crude chicken mortalities shows that poultry producers bear significant loss which lead to less profitability and termination of poultry business as well. In agreement to this, Hagos (2019) reported that disease-associated losses were the leading cause of poultry business termination in different areas of Tigray (North Ethiopia). In addition, our rough estimate of the economic impact only included the market price of the chicken as such, but did not consider eventual losses due to veterinary services costs, missed egg production, or nutritional value of the dead birds for the household. Given the high disease burden, more targeted studies assessing socio-economic impact of disease are thus needed.

The higher disease occurrence also clearly shows that it is challenging the achievement of the targets of the country’s poultry value chain masterplan (Shapiro et al. 2015) thereby compromising the sustainability of poultry business in Ethiopia. Similar to the present observation, Mazengia et al. (2012) reported a disease occurrence of 94% in the country. However, Addis et al. (2014) reported disease occurrence of 64.9% and 46% in small-scale intensive and extensive poultry production in Bahir Dar Zuria (Northwest Ethiopia). Sebho (2016) reported that diseases were the first frequently occurred chicken production constraints in Ethiopia.

Similarly, respiratory diseases, gastrointestinal diseases, eye-face-head diseases, and unidentified sudden death are the top four ranked diseases which could be considered as the leading causes of high chicken mortalities, morbidities, and monetary losses. Specific infectious diseases such as coccidiosis, fowl pox, salmonellosis, and flow cholera were also suggested to play negative roles on the growth and profitability of the sub-sector. In agreement to this, Asfaw et al. (2019) and Wubet et al. (2019) reported that diseases such as ND, coccidiosis, and others cause high morbidity and mortality in different production systems of Ethiopia. Similarly, Kebede et al. (2014) reported ND as the most frequently mentioned disease, followed by coccidiosis, fowl pox, and ecto-parasitism in North Gondar (Northwest Ethiopia).

We found that farmers with access to disease diagnosis and availability of animal health experts for chicken diagnosis reported higher disease incidence, which might reflect higher levels of disease awareness and an understanding that chicken can do better than what they currently do. Indeed, we found that less than half of farmers report having access to diagnostic services, and even less to diagnostic services by professional animal health experts. In line with this are the findings that less than 50% of household reported access to treatment for their chicken. We also found that households that used vaccination performed by non-professional chicken vaccinators had higher odds to report disease occurrence. Whether this is a true association or not is not possible to conclude, but highlights the need for thorough training of all vaccinators.

The high mean crude mortality (33.9%) signifies that more than one-third of the poultry population in the country are being killed every year due to the highly prevailing poultry diseases—most of which could be avoided by well-designed vaccination programs and better chicken health management. The high crude mortality seems to reflect the existing poor poultry health services as reported by others (Asfaw et al. 2019; Hooper 2016; Sambo et al. 2015). Another concern is that mortality rates were higher among women-owned chicken, which might reflect gender gaps in accessibility of diagnosis, treatment, and prevention, as seen for other agricultural activities of Ethiopia (Beesabathuni et al. 2019). The higher mortality in chicken flocks with absence of access to diagnosis and vaccination services might indicate that absence of diagnosis services contribute to delays in disease recognition and delay in treatment leading to death of more chickens. This finding is also in line with Hooper (2016) who reported that animal diseases in Ethiopia are neither diagnosed nor treated properly because cases are diagnosed only based on symptoms. Moreover, there are many vaccine-preventable poultry diseases prevailing in Ethiopia against which vaccines are not yet available in the country. In support of this, Beesabathuni et al. (2019) reported that poultry vaccines produced locally in Ethiopia are limited in number and volume, while imported ones take long waiting time and have potential biosecurity concerns.

The reported mortality is in line with Wubet et al. (2019) who reported a poultry mortality range from 20 to 50% in Ethiopia. Other studies reported even higher mortalities; Lemlem and Tesfay (2010) reported 68%, 48.5%, and 52% in different breeds in Northern Ethiopia. Similarly, Mazengia et al. (2012) reported higher overall mortality rate of 45% in day-old chickens in three agro-climatic zones of Amhara region (Northwest Ethiopia). However, the currently reported magnitude of the crude mortality is higher than the findings of Geleta et al. (2013) who reported lower mortality rate of 7.2% in Adami Tulu Research center. Similarly, Chanie et al. (2009) also reported an overall lower chicken mortality of 26.3%. The difference in the magnitude of the mortalities could be due to the variation in the poultry production system, time-frame, and the scope of the studies. The previously conducted studies are more or less small studies while the current study fairly represents the country and the magnitude of the crude mortality could be country-level representing finding.

The reported higher disease occurrence, their crude mortalities, and associated monetary losses are also a reflection of lack of disease control and prevention interventions, poor farm biosecurity and hygiene, and sub-standard ways of poultry keeping (Beesabathuni et al. 2019; Hagos 2019; Wubet et al. 2019). Moreover, lack of scientific knowledge and skills of poultry producers, low farm standards for minimum requirements (e.g., minimum healthcare, housing, and feeding and biosecurity standards), and lack of technical and institutional supports (Beesabathuni et al. 2019) acerbate the situation. While some attempts have been made to strengthen the private sector role in poultry health service delivery, more needs to be done in this regard (Shapiro et al. 2015). Despite its huge potential, the poultry sub-sector contribute less to solve the overwhelming poverty, malnutrition, stunting child growth, unemployment, lack of women and youth empowerments, low income, and the overall poor livelihood status of the poultry producers and the general public (Hagos 2019; Mazengia et al. 2012; Shapiro et al. 2015).

As to the conclusion, the high overall disease occurrence, means of crude chicken mortality, and monetary loss due to mortality are critical problems of the Ethiopian poultry sub-sector. These challenges are attributed to the existing weak poultry health service delivery system of the country. From the current findings, the authors recommend that the following five intervention areas (1) clarify roles and responsibilities of public and private sector poultry health service providers to ensure private sector can be competitive; (2) strengthen private poultry health service providers for rural, urban, and per-urban settings through public-private partnerships and improved business skills of service providers (develop novel poultry health service delivery modalities such as door-to-door health service provision and village-level poultry clinics run by private practitioners); (3) ensure needed poultry vaccines and drugs are available in the market; (4) build capacity of poultry producers on poultry disease control and prevention; and (5) define minimum standards for poultry housing, feeding, and biosecurity. These interventions not only would be a major step towards addressing current weaknesses of poultry health services but also require policies that are conducive to strengthen the poultry sector.

Finally, it is to be stated that due to technical reasons as well as the study areas were very wide to reach, the study had limitations to verify exact cause of the chicken’s sickness and deaths up on collection of biological samples from the chicken flocks and diagnosed them using laboratory tests. Hence, it is recommended that laboratory-based identification of the exact causes of chicken illness and deaths shall be the future research areas in Ethiopia.

## References

[CR1] Addis B, Tadesse D, Mekuriaw S (2014). Study on major causes of chicken mortality and associated risk factors in Bahir Dar Zuria District, Ethiopia. African Journal of Agricultural Research.

[CR2] Al Mamun, M., and Mehetazul, K. (2019). Occurrence of poultry diseases at Kishoregonj district of Bangladesh. *MOJ Proteomics & Bioinformatics* **8**.

[CR3] Asfaw Y, Ameni G, Medhin G, Alemayehu G, Wieland B (2019). Infectious and parasitic diseases of poultry in Ethiopia: a systematic review and meta-analysis. Poultry Science.

[CR4] Beesabathuni K, Lingala S, Kumari P, Otieno S, Olson R, Kraemer K (2019). Egg value chain Analysis in Etrhiopia, white paper.

[CR5] Chanie M, Negash T, Tilahun SB (2009). Occurrence of concurrent infectious diseases in broiler chickens is a threat to commercial poultry farms in Central Ethiopia. Tropical animal health and production.

[CR6] Charan J, Biswas T (2013). How to calculate sample size for different study designs in medical research?. Indian journal of psychological medicine.

[CR7] CSA (2018). Ethiopian Livestock population and characterstics.

[CR8] Ebsa YA, Harpal S, Negia GG (2019). Challenges and chicken production status of poultry producers in Bishoftu, Ethiopia. Poultry science.

[CR9] FAO (2019). Poultry Sector Ethiopia. FAO Animal Production and Health Livestock Country Reviews., Vol. 11. FAO, Rome.

[CR10] Geleta T, Leta S, Bekana E (2013). Production performance of Fayoumi chickens under intensive management condition of Adami Tulu research center. International journal of Livestock production.

[CR11] Habte, T., Amare, A., Bettridge, J., Collins, M., Christley, R., and Wigley, P. (2017). Guide to chicken health and management in Ethiopia. ILRI Manual 25. International Livestock Research Institute (ILRI), Nairobi, Kenya.

[CR12] Hagos (2019). Assessing Poultry Disease Diagnosis and Treatment Practices, and Cost of Diseases in Intensive Farms among Selected Zones, Tigray, Northern Ethiopia. MSc thesis., Mekelle Univeristy, Mekelle, Ethiopia.

[CR13] Hooper, P. (2016). Review of animal health service delivery in the mixed crop-livestock system in Ethiopia. LIVES Working Paper 18. . International Livestock Research Institute (ILRI), Nairobi, Kenya.

[CR14] ILRI-ACGG (2015). ACGG Site Selection Framework. African Chicken Genetics Gain (ACGG) Project. International Livestock Research Institute (ILRI), Nairobi, Kenya.

[CR15] Kebede H, Melaku A, Kebede E (2014). Constraints in animal health service delivery and sustainable improvement alternatives in North Gondar, Ethiopia. Onderstepoort Journal of Veterinary Research.

[CR16] Lemlem, A., and Tesfay, Y. (2010). Performance of exotic and indigenous poultry breeds managed by smallholder farmers in northern Ethiopia. *Livestock Research for Rural Development* **22**.

[CR17] Marangon S, Busani L (2007). The use of vaccination in poultry production. Revue Scientifique et Technique-Office International des Epizooties.

[CR18] Mazengia, H., Siraw, G., and Nega, M. (2012). Challenges and prospects of village-based exotic chicken development strategy in Amhara regional state, Northwest Ethiopia. *Global J. Sci. Front. Research. Agriculture. Veternary Science* **12**.

[CR19] Musa, L., Peters, K., and Ahmed, M. (2006). On farm characterization of Butana and Kenana cattle breed production systems in Sudan. *Livestock research for rural development* **18(12)**.

[CR20] Sambo E, Bettridge J, Dessie T, Amare A, Habte T, Wigley P, Christley RM (2015). Participatory evaluation of chicken health and production constraints in Ethiopia. Preventive veterinary medicine.

[CR21] Sebho HK (2016). Exotic chicken status, production performance and constraints in Ethiopia: a review. Asian Journal of Poultry Science.

[CR22] Sergeant, E. (2009). Epitools epidemiological calculators. Ausvet. Available at: http://epitools.ausvet.com.au. AusVet Animal Health Services and Australian Biosecurity Cooperative Research Centre for Emerging Infectious Disease, Australia.

[CR23] Shapiro, B., Gebru, G., Desta, S., Negassa, A., Nigussie, K., Aboset, G., and Mechal, H. (2015). Ethiopia livestock master plan. *In* "ILRI Project Report. Nairobi, Kenya: International Livestock Research Institute (ILRI)".

[CR24] Wubet W, Bitew M, Mamo G, Gelaye E, Tesfaw L, Sori H, Zewdie T, Abayneh T (2019). Evaluation of inactivated vaccine against fowl cholera developed from local isolates of Pasteurella multocida in Ethiopia. African Journal of Microbiology Research.

